# Orofacial Cleft and Mandibular Prognathism—Human Genetics and Animal Models

**DOI:** 10.3390/ijms23020953

**Published:** 2022-01-16

**Authors:** Anna Jaruga, Jakub Ksiazkiewicz, Krystian Kuzniarz, Przemko Tylzanowski

**Affiliations:** 1 Laboratory of Molecular Genetics, Department of Biomedical Sciences, Medical University of Lublin, Chodzki 1, 20-093 Lublin, Poland; annajaruga@umlub.pl (A.J.); kubaksiazkiewicz@gmail.com (J.K.); 2 Center for Molecular and Vascular Biology, Department of Cardiovascular Sciences, University of Leuven, Herestraat 49, 3000 Leuven, Belgium; 3 Department of Maxillofacial Surgery, Medical University of Lublin, Staszica 11, 20-081 Lublin, Poland; krystiankuzniarz@umlub.pl; 4 Department of Development and Regeneration, University of Leuven, Herestraat 49, 3000 Leuven, Belgium

**Keywords:** cleft lip and palate, prognathism, candidate genes, zebrafish, chicken, mouse

## Abstract

Many complex molecular interactions are involved in the process of craniofacial development. Consequently, the network is sensitive to genetic mutations that may result in congenital malformations of varying severity. The most common birth anomalies within the head and neck are orofacial clefts (OFCs) and prognathism. Orofacial clefts are disorders with a range of phenotypes such as the cleft of the lip with or without cleft palate and isolated form of cleft palate with unilateral and bilateral variations. They may occur as an isolated abnormality (nonsyndromic—NSCLP) or coexist with syndromic disorders. Another cause of malformations, prognathism or skeletal class III malocclusion, is characterized by the disproportionate overgrowth of the mandible with or without the hypoplasia of maxilla. Both syndromes may be caused by the presence of environmental factors, but the majority of them are hereditary. Several mutations are linked to those phenotypes. In this review, we summarize the current knowledge regarding the genetics of those phenotypes and describe genotype–phenotype correlations. We then present the animal models used to study these defects.

## 1. Introduction

The development of craniofacial structures is a complex process regulated by several signaling pathways including bone morphogenetic proteins (BMPs), Sonic hedgehog (Shh), and Wnt [1,2]. Mutations affecting these pathways frequently result in craniofacial abnormalities and associated malocclusion including orofacial clefts and prognathism [3,4]. Anatomically, clefts (CL/P) are classified as a cleft lip and/or palate or an isolated cleft palate with unilateral or bilateral variations. They may also occur as a part of syndromic disorders. The clinical manifestations of CL/P are variable because of the many genes involved. Genes frequently associated with CL/P include *IRF6*, *TBX22*, *MAFB*, *ARHGAP29*, *VAX1*, and *PAX7* [5,6]. Prognathism, also called class III malocclusion, is defined as an abnormal forward projection of the mandible beyond the standard relation to the cranial base with or without hypoplasia of maxilla. The molecular basis of prognathism is unclear. Numerous studies focused on the inheritance of MP have been inconclusive, but several candidate genes have suggested that the etiology is most likely polygenic and multifactorial [7].

### Biology of Palate Development

The palate separates the oral and nasal cavities and can be classified into hard and soft ones. Anteriorly, the palate consists of a bony structure, and posteriorly, it consists of a muscular and soft structure. The soft palate additionally acts as a flap that closes nasal airways during swallowing and regulates airflow while speaking. The developmental origin of the structure is mixed, with both neural crest and paraxial mesoderm contributions [8]. During embryonic development, primary and secondary palates are formed. The primary palate consists of the philtrum and upper incisor region of the upper jaw anteriorly to the incisive foramen. It is derived from frontonasal prominence, which is a part of the rostral boundary of a primitive mouth. The secondary palate includes every other part of the hard and soft palate emerging from outgrowths placed at the oral side of the paired maxillary prominences located on both sides of the primitive mouth. These outgrowths expand vertically, flank the developing tongue, and become palatal shelves. Later, palatal shelves horizontally change their position to grow above the tongue. Following re-orientation, they grow towards each other and fuse with themselves, as well as the primary palate and nasal septum, to form the complete roof of the oral cavity [8]. Shh, secreted by oral epithelial cells, promotes the growth of palatal shelves [9] by maintaining the expression of transcription factors such as *Foxf1*, *Foxf2*, and *Osr2* that control the elongation of palatal shelves [10].

At a specific moment, palatal shelves horizontally change their orientation and elevate above the tongue. Multiple hypotheses have been put forward to provide insight into the mechanisms regulating the elevation of palatal shelves. The most popular one suggests that hyaluronic acid (HA), an extracellular molecule binding large amounts of water, concentrates at higher levels in specific regions of palatal mesenchyme and drives osmotic pressure to cause remodeling movement of palatal shelves. This hypothesis is supported by data showing that biotin-labeled HA binding peptides have different localizations than HA in the palatal mesenchyme. Additionally, mice lacking Golgi-associated protein GOLB1 have reduced HA concentration in the mesenchyme of the palate concomitant with the failure of palatal shelf elevation, supporting this notion [11]. However, the source of “internal force” that causes palatal shelves to elevate remains elusive.

A recent study suggested that the re-orientation of middle and posterior palatal regions is induced by actin-based cell contraction together with changes in extracellular matrix (ECM) composition, thus increasing rigidity within palatal shelves [12], but further research is required to answer how these processes can affect palate development. Besides HA, numerous other ECM components are present in forming palatal structures such as tenascin-C and tenascin-W. *Foxf2* null mice showed the failure of palatal shelf elevation, the significantly reduced expression of various ECM molecules including tenascin-C and fibronectin, and a low number of ECM integrin-β1 receptors [13]. During elevation, OSR2 and PAX9 transcription factors are present along the medial–lateral axis, and *Osr2*^−/−^ and *Pax9*^−/−^ mice have defective palatal shelf elevation [14]. *Ldb1*^−/−^ mice show reduced *Osr2* and *Pax9* expression in developing mesenchyme with similar phenotypes [15].

The Wnt–planar cell polarity (PCP) pathway is also involved in palatal shelf elevation. Mice lacking *Wnt5a* and/or its receptor *Ror2* display incorrect palatal shelf elevation phenotypes [16]. PCP is regulated by small GTPases such as RAC1, and a recent study showed that levels of this protein were regulated in the palatal mesenchyme prior to palatal shelf elevation; additionally the overexpression of *Rac1* was found to lead to disruptions in palatal shelf reorientation [1]. These data support the role of the Wnt–PCP pathway during palatogenesis.

The next step in palatogenesis is the fusion of palatal shelves above the tongue to form the complete roof of the oral cavity. Mice null for *Jag2*, *Irf6*, *Grhl3*, or *Fgf10* have displayed cleft palates and abnormalities in the fusion process [17,18,19,20]. These data, together with analyses of the double *Irf6/Jag2* and *Irf6/p63* mutants, have revealed a molecular network involving *Fgf10/Fgfr2* and *Jag2/Notch* signaling regulating the process of periderm formation, a monolayer of flat epithelial cells that covers the internal and external surfaces of the embryo during development [21]. The genetic ablation of periderm cells is the cause of dysfunctional oral epithelial adhesions. This finding suggests that the periderm acts as a barrier that prevents pathological epithelial adhesions [22], suggesting that the periderm needs to be removed during the fusion of palatal shelves. The apoptosis of peridermal cells during palatal fusion has reported, though molecular events controlling this phenomenon are not fully understood [23]. TGFβ3 signaling appears to have a crucial role in those events [8]. For instance, mice lacking TGFβ3 fail palatal fusion. Placing these palates in explant cultures, even with direct contact, fails to rescue the fusion, potentially because *Tgfβ3*^−/−^ embryos have palatal shelves that are still covered with persistent peridermal cells.

## 2. Development of the Mandible

The mandible is the largest bone of the human skull and, like most of the palatine structures, is of neural crest origin. It originates from the first branchial arch (BA), which can be divided into the mandibular and maxillary processes. The expression of genes from the distal-less (Dlx) family is crucial for this distinction and defines the boundaries between mandibular and maxillary processes. Dlx1 and Dlx2 are ubiquitously expressed within the first BA, whereas the expression of Dlx3-6 is restricted to specific distal parts of the mandibular process. Dlx5 and Dlx6 reach into the regions of the future hinge between the mandible and the maxilla. In contrast, the expression of Dlx3 and Dlx4 is even more restricted to the most distal region of the mandibular process, being controlled by Dlx5 and Dlx6 [24]. The function of Dlx genes in mandible specification was confirmed in vivo, when the loss of function of Dlx5 and Dlx6 led to the homeotic transformation of the mandible into maxilla.

Following the migration of cranial neural crest cells, mandibular prominence is patterned along both the proximal–distal and oral–aboral axes. The epithelium plays an important role in this process, similarly to the patterning of palatal shelves. In the case of the proximal–distal pattern, the key pathways are BMP and FGF. Both act antagonistically and therefore limit the expression of target genes to a specific regions. *Fgf8* is expressed in the proximal domain of the mandibular ectoderm, while *Bmp4* is in its distal domain. The overexpression of Bmp4 within the cranial neural crest leads to the bony fusion of the maxilla and mandible and the hypoplasia of the mandible and cleft palate [25], while the inhibition of BMP within the developing mandibular arch alone causes the change in tooth shape from incisor to molar.

Additionally, FGF is involved in determining the patterning on the oral–aboral axis. The distribution of cranial neural crest cells in oral mesenchyme overlaps with the expression of the *Lhx6* and *Lhx8* genes. On the aboral side, *Goosecoid* (*Gsc*) is actively expressed in the absence of *Lhx6/8*. This allows the mandibular arch mesenchyme to be divided into *Lhx6/8*-positive and *Gsc*-positive regions, with *Fgf8* inducing *Lhx6/8* expression and repressing *Gsc* expression. A recent study using scRNAseq revealed that *Shh* expression is restricted to the oropharyngeal epithelium. The inactivation of *Shh* within this tissue leads to tongue agenesis, hypoplasia, and the distal truncation of the mandible [26]. The data show that the SHH expressed in oropharyngeal epithelium antagonizes BMP signaling and patterns the oral–aboral axis of the mandibular arch [25]. This type of epithelial–mesenchymal interaction involving Shh corresponds to the situation in developing palates, where the expression of *Shh* in epithelium drives the outgrowth of mesenchymal-derived palatal shelves.

The next step in mandibular development is the emergence of Meckel’s cartilage (MC). MC is an intermediate step for the development of the mandible [26,27]. Initially, it appears as the region of condensed mesenchymal cells near where the first molar would appear. The mesenchymal cells differentiate into chondrocytes and form bilateral cartilage tissue, which elongate both anteriorly and posteriorly to fuse at the most distal part of the mandibular arch. MC acts as a mechanical reinforcement prior to the mandibular ossification [28]. MC can be subdivided into three regions: distal, intermediate, and proximal. The distal part will give rise to the intramandibular symphysis, while the proximal region give rise to the inner ear ossicles. The intermediate region disappears shortly after birth. The exact mechanism for the degradation of this region has not been fully described, but is known that the processes of autophagy and macrophage activity are vital [29,30].

The mandible ossification process is not uniform, with the distal and proximal regions formed by endochondral-like processes and the intermediate region formed by intramembranous ossification. Symphysis development in the distal part is strongly dependent on *Ihh* signaling, and mice lacking *Ihh* show developmental defects of symphysis involving enhanced chondrocyte maturation and the decreased proliferation of chondroblast progenitors [31].

## 3. Orofacial Cleft—Clinical Features and Manifestations

The cleft of the lip with or without cleft palate (CL/P) is the most common congenital disorder of the craniofacial region in humans. Depending on the geographical location and the ethnic group, CL/P is diagnosed in 1–7 per 1000 births [32]. Additionally, the predilection for CL/P is greater for males, while the frequency of CP is similar for both genders [33]. The phenotype is characterized by a lack of connection between hard and/or soft tissues within the orofacial region. The first branchial arch plays a key role in the development of the oral cavity. Disturbances at the specific stages of fetal development, including upper lips and maxilla formation, determine the appropriate and specific phenotype of a cleft. The development of a cleft lip and/or alveolar process occurs in a period of 4–6 weeks of fetal life during the formation of the primary palate [32,34,35]. During that time, mesoderm projections migrate from the first branchial arch into the jaw area surrounded by two epithelial walls. The delay of this process or the lack of at least one of these three tissue contributes to branchial membrane cleaving. Depending on the position and number of lost mesoderm components, it causes unilateral, bilateral, or median fissure [36]. The subsequent formation of the secondary palate proceeds between 6 and 10 weeks of fetal life [32,34,35]. Palatal shelves play pivotal roles in palatogenesis through mesodermal processes covered by the ectoderm that medially migrate and join starting from incisive foramen and then posteriorly towards the throat as a secondary palate [36,37]. Palate clefts are caused by disorders taking place during this period of development, with broad clinical manifestation ranging from a mild cleft of the uvula or by a complete cleft of the hard and soft palate [36]. They can occur as an isolated disorder (CP) or coexist as a part of a syndrome. Moreover, they can be observed in unilateral or bilateral forms [38]. Interestingly, unilaterally occurring defects are twice as frequent on the left side [5,39]. These anomalies pose challenges for not only the patient and the family but also the therapeutic team due to the improper functioning of the craniofacial apparatus, which affects each element of the stomatognathic system. Specifically, a lack of developmental connection between the tissues of the lip, alveolar process, and potential oronasal fistula may affect such activities as sucking, swallowing, the expression of emotions, the formation of thoughts, and efficient communication [40,41,42]. Moreover, the impaired formation of negative intraoral pressure during sucking and swallowing in this disorder results in the impaired nutrition of the newborn and an unsatisfactory pace of weight gain [41]. Nevertheless, clefts may also contribute to numerous disorders of jaws, e.g., hypodontia, malocclusion, and hypoplasia of the maxilla [43]. The coexisting underdevelopment of the upper jaw is caused by increased soft tissues tension within a cleft area that is related to intrinsic tissue deficiency, with scarring being a result of surgical procedures and the overactivity of orbicularis oris muscle [44]. Delay in dental development, as well in terms of tooth eruption into the oral cavity, are the most frequent dental complications noticed in CL/P patients. The average delay was evaluated at 6–7 months. Moreover, the asymmetric development of the dentition causes a delay in the onset of combined orthodontic and surgical treatment and thus intensifies other functional disorders related to this anomaly [45].

According to the classification described in the International Perinatal Database of Typical Oral Clefts, these disorders can be divided into nonsyndromic CL/P and syndromic CL/P [46]. In clinical practice, diagnosing syndromic CL/P (SCL/P) requires the presence of at least two additional symptoms not related to craniofacial development. On the contrary, nonsyndromic CL/P (NCL/P) is observed as an isolated, non-specific form or if additional malformations are unrecognizable [47]. Clefts may be a part of clinical features in over 400 genetic syndromes [48,49]. Nonsyndromic forms of CL/P are more common, and they account for 93–95% of cases [48]. Recent studies have shown that the prevalence of a cleft lip with or without plate ranges from 3.4 to 22.9 per 10,000 births. In turn, the isolated cleft palate was diagnosed in 1.3–25.3 per 10,000 births. The frequency of cleft defects depends on the geographical region and ethnic group. China, Japan, and parts of Latin America are areas where CL/P is more frequently diagnosed. However, this anomaly is rarely observed in Israel, South Africa, or Southern Europe. Moreover, the occurrence of CP was found to be higher in Canada and parts of Northern Europe, as well as lower in parts of Latin America and South Africa. It should also be noted that the migration process has not changed rates of clefts occurrence within different ethnic groups in the UK and USA [32]. In addition, American Indians are characterized by the highest rate of NCL/P occurrence (3.6:1000) in comparison to Asians, Whites, and Afro-Americans.

The first report indicating the effect of genetic factors linked to NCL/P incidence was published in 1942 by Fogh-Anderson. A correlation was observed between the frequency of cleft occurrence in families where the disease appeared in previous generations [50]. Moreover, Leslie et al. proposed that the risk of this disease is 32-fold higher in patients with a positive pedigree history [5]. The identification of genes responsible for NCL/P will result in understanding the molecular events leading to this phenotype. Although a wide range of candidate genes and risk loci have been associated with clefts formations, no single locus or group of loci have been unequivocally identified with the phenotype (Table 1). However, most of the literature data support *IRF6*, *MAFB*, *ARHGAP29*, 8q24, *VAX1*, and *PAX7* as candidate genes [5]. It was noted that *IRF6* is the most frequently detected in the Asian group, while 8q24 is the most frequently detected in the European group. Other reports suggest a possible interaction between *MLLT3* and *SMC2* on chromosome 9 and alcohol consumption during pregnancy. A similar relation was noted among *TBK1* on chromosome 12 and *ZNF236* on chromosome 18 and smoking. Moreover, a deficiency of multivitamin supplementation during pregnancy reflects mutations within *BAALC* on chromosome 8 [33]. Genome-wide meta-analyses of nonsyndromic orofacial cleft phenotypes conducted among cross-ethnic group revealed two novel candidate genes that—when affected—may lead to congenital malformations. The study showed correlation between SNP rs76479869 detected within the third intron of *TP63* and the CL/P phenotype. *TP63* is a well-known transcription factor involved in epidermal morphogenesis. However, numerous syndromic skeletal disorders, e.g., ectrodactyly–ectodermal dysplasia clefting syndrome, split-hand/foot malformation, and limb-mammary syndrome, are associated with a dominant mutation of *TP63*. In addition, the unique association between SNP rs12347191 within 9q22 near *FOXE1* and all types of orofacial clefts was confirmed. Previous studies linked the recessive mutation of *FOXE1* with Bamforth–Lazarus syndrome, which is described as cleft palate and congenital hypothyroidism. Moreover, the results of animal studies have shown that mice with a missense of *FOXE1* were diagnosed with cleft palates and the abnormal development of thyroid [1] Interestingly, rare and damaging mutations lead to syndromic orofacial cleft forms that are subject to monogenetic or chromosomal inheritance. The more common and less destructive forms can phenotypically manifest as nonsyndromic, even if they involve the same gene, such as in the Van der Woude syndrome; this is the most common syndrome that is accompanied by CL/P in 15% of patients [5] and linked to mutations in the *IRF6* gene (locus 1q32-q41). It is inherited in an autosomal dominant pattern with a penetration of 89–99% and is responsible for 2% of all cleft cases. It occurs with the highest frequency in Caucasians (0.81 per 1000 births) compared to Asians (0.76 per 1000 births), Hispanics (0.74 per 1000 births), and Blacks (0.41 per 1000 births) [51]. Besides the occurrence of cleft, it is characterized by the presence of the lower lip mucoceles or cysts and hypodontia [5,35,48,52]. Syndromic forms of orofacial clefts may be also noticed within other disorders, e.g., Pierre-Robin Sequence, Treacher-Collins Malformation, Trisomies 13 and 18, Apert’s Syndrome, Stickler’s Syndrome, and Waardenburg’s Syndrome [53,54,55,56]. Comparing the inheritance character of NCL/P with SCL/P, it is worth noting that CP is more frequently related to genetic syndromes than CL/P [57].

Recent large-scale analyses combining the results of previous selected GWAS investigations on nonsyndromic forms of CL/P and CP have provided numerous significant conclusions. 2p21_PKDCC_, 14q22, 19p13, and 15q24 have been recognized as new loci involved in the etiopathology of CL/P. Moreover, potential candidate genes located within 2p21 and 14q22 have been also identified, e.g., proteins playing key roles in the orofacial embryogenesis of zebrafish and mice—PKDCC, FMRD6, and TMX1. However, the research results for CP have not been as satisfactory compared to those for CL/P. An evaluation of GWAS data using of polygenic score approach excluded a hypothesis about different alleles having the same effect on CL/P and CP development, thereby confirming the different molecular basis of both cleft forms. The authors hypothesized that CP is associated with rare or low-frequency variants in comparison to common variants in non-coding regions that contribute to CL/P [2].

The broad range of clinical manifestation of CL/P is likely caused by the variable expression of genes. In contrast to the incomplete form, the complete one has a cleft of the lip covering a full range of tissues and is commonly related to a cleft placed within the maxilla alveolar process [38]. One of the possible forms of a complete cleft in Simonart’s band, which is described as a band composed of the various volumes of soft tissue placed between the medial and lateral portions of a lip or within the fissure of the upper jaw alveolar ridge [65]. This tissue band may be formed by skin, blood vessels, nerves, mucosa, and distinct numbers of orbicularis oris muscle fibers [38,65]. Due to asymmetric growth, a unilateral cleft lip causes numerous esthetic and functional disorders that can be partially reduced by an inherency of Simonart’s band. The curvature of the thin vermillion border (known as the white roll) and the dislocation of the philtrum, in this case, can be observed on the cleft side. The peak of the Cupid’s bow is turned superiorly as well. Moreover, a complete lack of the philtral ridge on the pathologically changed side and the flattening of the philtral dimple is marked. A distinctly decreased volume of orbicularis oris muscle fibers was observed on the affected side in comparison to the contralateral side [66,67]. Additionally, the physiological positions of orbicularis oris muscle fibers are also disrupted. Thus, the lateral part of the muscle runs parallel to the fissure and laterally fuses with the nasal ala cartilage, and the medial component attaches to the columella [67]. Additionally, ipsilateral nostrils may also be influenced by cleft distortions where the dislocation of the lower lateral cartilage of external nose laterally, inferiorly, and posteriorly may cause horizontal and flattened profiles [68]. On the contrary, a tip of the external nose is inferiorly displaced and in the direction of the healthy side. Due to the lack of continuity of piriform foramen borders, the length of a columella is distinctly decreased and deflected to the non-affected side [66,68]. Moreover, the nostril on the affected side may be wider and the dome of the external nose may be far more flattened than the non-affected side [69].

In contrast to unilateral clefts, the symmetrical and complete defects are characterized by a lack of connection between premaxilla (called also prolabium) and both maxillae. Thus, single malformations appear as a fusion of the prolabium and upper jaw only on one side. These differences affect the direction and dynamics of the prolabium growth process. In single clefts, it develops towards the physiological side with disfiguring rotation and distinct asymmetry. On the contrary, in double clefts, the premaxilla grows apart from the rest of the upper jaw. Despite its forward position, symmetry is predominantly retained. In addition, its characteristic feature is a lack of orbicularis oris muscle fibers [67]. Clinically, the physiological shape of the upper lip is deformed due to the abnormal formation of Cupid’s bow and both philtral ridges. In comparison to unilateral defects, the columella is hypoplastic or completely absent, with the lateral crura laterally moved and resulting in alar flaring [38]. The appearance of both nostrils is identical to clinical features of a unilateral cleft. In turn, the cleft observed among primary palate manifests as a groove from incisive foramen to the alveolar process of the maxilla, and it constantly appears with cleft of the lip. In contrast, clefts of the secondary palate are noted as submucosal or complete gaps within hard and/or soft palates. Therefore, a differential connection level between the residual palate and vomer representing a component of nasal cavity septum can be observed.

Defects within the soft palate fusion lead to disorders of palatal musculature anatomy since muscle fibers attach to the posterior margin of the hard palate [70]. It should be noted that microforms or subclinical features have been recognized, e.g., congenital healed cleft lip, defects of the orbicularis oris muscle, and lip pits/prints [33,71]. A lack of typical manifestations of these disorders requires that clinical examinations in these cases is typically supplemented using high-resolution ultrasound due to the unaffected, superficially visible tissues [71]. Due to the distinct differences in phenotype, they have not been classified as CL/P, but they may present the milder risk gene expression responsible for CL/P [33]. Furthermore, subclinical phenotypes of cleft palate may also be noticed, but they have been less discovered compared to CL/P. Literature data describe these anomalies as a bifid uvula, midline diastasis of the velar musculature, ankyloglossia, or even a notch place in the posterior margin of the hard palate [33,71]. Submucosal clefting within the hard and/or soft palate may be observed as well. Unfortunately, due to the potential risk of orofacial fistula, they may contribute to more complex functional disorders compared to both cleft lip and maxilla alveolar ridge, e.g., feeding difficulties, velopharyngeal insufficiency, and speech problems [38].

## 4. Mandibular Prognathism (MP)—Clinical Features and Manifestation

The occlusion status of patients is defined by the three-level Angle’s classification describing sagittal dental relation based on co-position of the first molars of both jaws. Physiological occlusion (class I) is manifested by a natural contact between the mesiobuccal cusp of the upper first molar and the buccal groove of the mandibular first molar. An anterior shift of the same cusp to the buccal groove of the lower first molar indicates a retrusion of mandible that is classified as class II [72]. Class III malocclusion occurs when the mesiobuccal cusp of the upper first permanent molar is distally placed to the mesiobuccal grove of the lower first permanent molar [73]. This skeletal deformity or mandibular prognathism (MP) is one of the most frequent dentofacial anomalies. It is responsible for 63–73% of all class III malocclusions [74], commonly known as a Habsburg jaw, it is defined as disproportional overgrowth of mandible in relation to the rest of craniofacial skeleton (particularly to the cranial base), and it may coexist with or without the hypoplasia of maxilla [75,76]. The incidence of MP within ethnic groups varies, with the highest ratios in Asian populations (15%) and only 1% in Caucasian populations [77]. Some studies have also reported on African populations, with a prevalence between 10% and 16.8% [76]. Besides cosmetic defects, MP can lead to serious functional disorders reducing the quality of life. Affected patients predominantly complain about inconveniences with mastication, speaking, and pronunciation. Additionally, it may decrease self-confidence and social skills, and then it may consequentially cause problems in the social relationships of patients as well [78].

**Figure 1:**
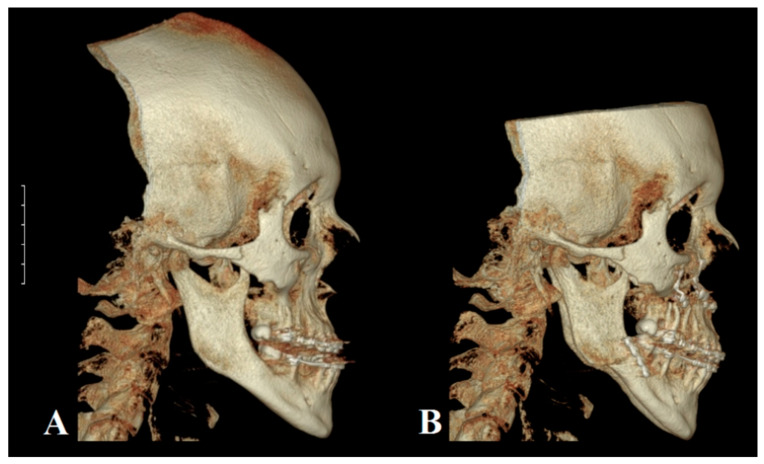
Representative 3D reconstructions of cone beam computed tomography (CBCT) examinations. Patient was diagnosed with class III skeletal malocclusion. (**A**) CBCT performed during pre-surgical orthodontic treatment. (**B**) Postsurgical treatment CBCT. The Le Fort I osteotomy of maxilla and the bilateral sagittal split osteotomy of mandible have been performed. The protrusion of maxilla and the retrusion of mandible were conducted to restore proper occlusal status. Osteosynthesis elements are visible (white) within both jaws.

**Figure 2:**
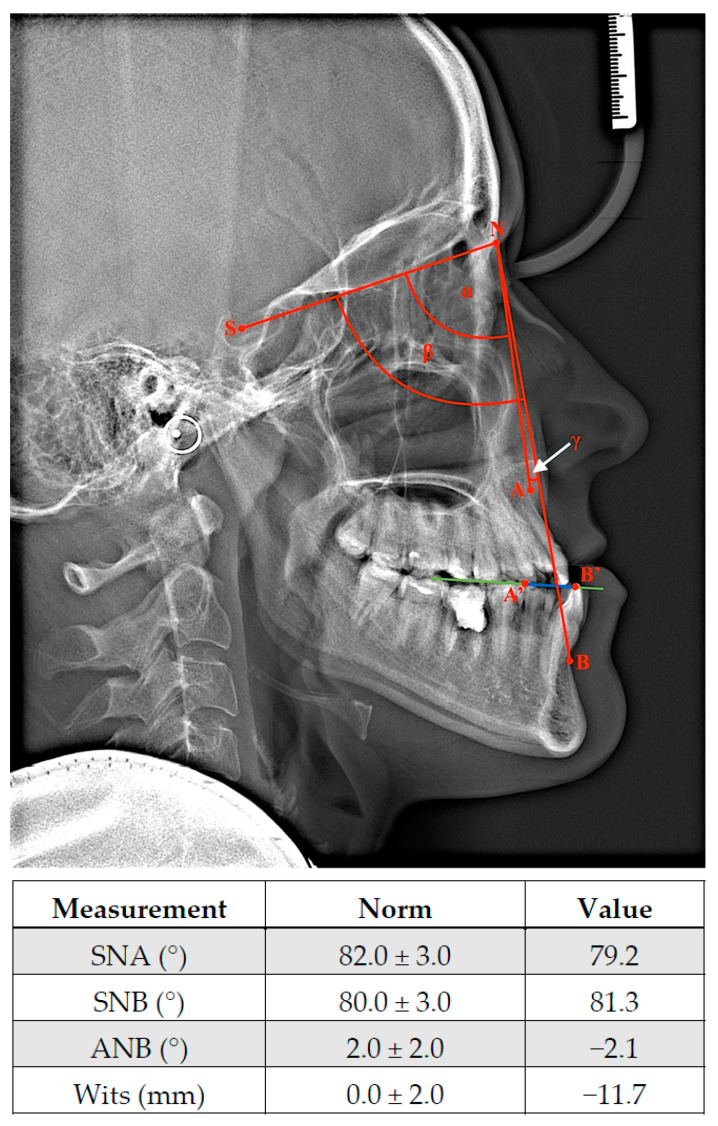
Representative cephalometric X-ray and part of cephalometric analysis according to Segner and Hasund presenting patients diagnosed with skeletal class III malocclusion before treatment. A-SNA; β-SNB; γ-ANB (white arrow); A’-B’0Wits (blue line); green line-occlusal plane. See the details description in text and Supplementary Materials section (Table S1) for all cephalometric measurements.

Morphometric analyses of facial profile provide information about potential gnathic defect in patients. Specifically, the concave profile, protrusion, and extension of the lower facial area are measured [79]. In extreme cases, long face syndrome can appear as part of the class III malocclusion phenotype [80], where the relationship between the upper and lower lip is distinctly disturbed. Due to an average 2 mm retrusion and potential atrophy of the upper lip, the eversion of the lower lip, difficulties with proper lip contact, or even a lack of connection between them may be observed [81]; these additionally reduce chin prominence and decreasing of depth of labiomental fold [80]. MP is manifested on the midface, and the paranasal area, nasolabial fold, and cheek line are flattened [7]. Due to the complex nature of the stomatognathic system, each growth irregularity forces changes within teeth position and thus occlusal status. During intraoral inspection, angle class III and canine class III with negative incisional overjet and reduced overbite are observed [74]. Moreover, open bite (anterior or complex) and cross bite (unilateral or bilateral) may be accompanied in the clinical image of MP. Interestingly, the occurrence of any dentofacial abnormalities leads to the activation of a compensatory mechanism aimed to restore functional balance within the orofacial region. Therefore, lower incisors are predominantly retroclined against proclined maxilla incisors. Additionally, modifications of the symphysis region and increased incisor eruption level may disproportionately compensate within the vertical dimension [82]. The potential coexistence of maxilla hypoplasia with MP may aggravate the abovementioned symptoms and clinical manifestations of class III skeletal malocclusion due to the posterior position of the upper jaw (Figure 1). Isolated maxilla deficiency can phenotypically provide similar features despite correct physiological mandibular proportions. It is worth noting that the skeletal conditioning of class III malocclusion may be ethnically different. It has been reported that in US population class III malocclusion mostly occurs as a maxillary hypoplasia and protrusion. In contrast, in Asians, this deformation is predominantly diagnosed with a normal upper jaw and the overgrowth of the mandible [83,84]. To avoid the misdiagnosis of improper upper jaw or mandible development, a lateral cephalometric radiograph with cephalometric analysis should be performed [84]; this is a standardized orthodontic method and a part of the diagnostic process of malocclusion and treatment planning. Angular and linear measurements carried out on a radiograph allow for the assessment of the positions of various anatomical structures [85]. Despite only analyzing skeletal relationship in the sagittal plane, this strategy determines which anomalies, dental or skeletal, underlie the disorder [86]. Angular and linear measurements carried out on a radiograph allow one to assess the anterior or posterior position of both jaws in relation to the anterior cranial base (Figure 2) [87]. A position on a cephalometric X-ray is marked by a line running from point S (midpoint of sella turcica; sella) to N (anterior end of nasofrontal suture, nasion). In turn, the localizations of the maxilla and mandible is indicated by point A (the most posterior point on the premaxilla in the midline and below anterior nasal spine; Subspinale) and point B (the deepest point in a fossa above chin; Supramentale), respectively [88]. Therefore, the value of angle between the SN and NA planes provides information regarding the sagittal position of the upper jaw in reference to the cranial base (82 ± 3°). In addition, the size of the angle created by the SN and NB allows one to lines assess the antero–posterior position of the mandible in comparison to the anterior part of the skull base (80 ± 3°) [89]. Both angles are recommended for measurements of retrognathism and MP in clinical practice [87]. Moreover, the evaluation of the mutual locations of mandible and maxilla plays a pivotal role in diagnosis and treatment planning as well. This relationship shows an ANB angle with a value for class I malocclusion from 0° to 4°. Thus, class II and III may be diagnosed when the value of ANB is above 4° and less than 0°, respectively. It is noteworthy that Wits appraisal, as a distance between point A and B projected onto an occlusal plane, may be also valuable for proper malocclusion diagnosis [90]. Physiological class I is in the range from −2 to 2 mm. Class II and III have Wits values of over 2 mm and less then −2 mm, respectively [91]. All of the abovementioned values have been presented according to Segner–Hasund’s cephalometric analysis [92].

## 5. Mandibular Prognathism—Genetic Background

Numerous studies have focused on the inheritance of MP but have so far been inconclusive. Most likely, etiology is polygenic with a multifactorial background [7]. Some authors reported a single autosomal dominant inheritance pattern, but partial penetrance and variable manifestation environment factors should be considered as well. Additionally, the occurrence rate of MP was found to be six times higher within monozygotic twins than dizygotic twins [93]. Moreover, numerous mouse genetic studies have suggested that different mutations may only result in defects in selected anatomical regions. 

Linkage analysis may be a useful method in the analysis of MP inheritance, but its results are of limited value since they only indicate an approximate position of defective genes within evaluated loci. One of the first studies performed on Korean/Japanese and Hispanic families suggested the importance of loci 1p36, 6q25, 19p13.2 and 1p22.1, 3q26.2, 11q22, 12q13.13, and 12q23 in MP inheritance [93,94].

The developmental trajectory of mandibular development suggests that mutations localized within 1p36, as well as those containing genes responsible for skeletal development, may play pivotal roles in the pathogenesis of MP. Two of those genes, *ALPL* (alkaline phosphatase) and *HSPG2* (heparan sulfate proteoglycan 2), are considered to be candidate loci involved in the etiology of this disorder [94]. Numerous mutations have been identified in the *ALPL* gene in groups of patients diagnosed with hypophosphatasia, thus confirming its involvement in the bone mineralization process [95,96]. *HSPG2* expression is involved in bone marrow, skeletal muscle, and (importantly) cartilage development [97]. Additionally, studies have demonstrated the influence of polymorphism of *MATN1* (cartilage matrix protein) and *EPB41* (erythrocyte membrane protein band 4.1) on increased MP occurrence [98,99]. The mandibular process is the result of two types of bone formation—intramembranous and endochondral ossification [100]. Thus, the expression of *MATN1* within forming cartilage supports the relationship between polymorphism and higher malocclusion occurrence [101,102]. Interestingly, *EPB41* encodes the protein responsible for the morphology and structural stability of the red cell plasma membrane. Association analyses performed among Chinese patients diagnosed with MP indicated fours SNPs in *EPB41* that may be involved in the pathogenesis of class III skeletal malocclusion: Rs2249138, rs2254241, rs2788890, and rs2788888 presented significant variations in genotype distributions and allele frequencies. However, despite the relevance of the obtained results, the authors confirmed evident limitations due to conducting the investigation within only one ethnic group, so the mechanism and function of this gene in the etiology of MP remain unclear and require further studies [99].

Chromosome 12q13 contains *HOX3* and *COL2A1* loci, which considered to be potential candidate genes in MP pathology [94,103]. *COL2A1* (collagen type II, alpha 1), a cartilage marker, is located between 12q13.11 and 12q13.2. Studies have confirmed its influence on craniofacial growth as well [104]. Moreover, Xue et al. reported on the relation between *COL2A1* gene polymorphism and the MP phenotype [103]. The *HOX3* region is a part of the *HOX* complex; it includes at least seven genes, and it is involved in the patterning of the hindbrain and craniofacial structures [104,105]. Nevertheless, the status of 12q23 loci is still unresolved. For instance, the *IGF1* gene localized within this region encodes an insulin-like growth factor 1, and it has been perceived as the best possible candidate gene in this loci [7,106]. Receptors of this factor were noticed in the mandible condylar process, and researchers have disclosed its role in the determination of body size [107]. However, there have been other studies that found no association between *IGF1* and MP [103].

The research performed by Guan et al. deserves special attention. In a large family of 21 people where 9 individuals were affected by MP, one nonsynonymous single-nucleotide missense mutation (c. 742I > T) was found within *ADAMTS1* (a disintegrin and metalloproteinase with thrombospondin motifs 1). Moreover, it was shown that that two single-nucleotide polymorphisms (rs2738 and rs229038) within *ADAMTS1* were linked to MP [108]. The involvement of *ADAMTS1* in MP was confirmed in an investigation conducted by Kantaputra et al. Two unique mutations, i.e., c.176C > A and c.670C > G, within four families were observed. Interestingly, two out of four individuals in Family 2 had supplemental *COL1A2* mutations and were additionally diagnosed with osteogenesis and dentinogenesis imperfecta. Furthermore, a *WNT10B* mutation was discovered in a case of Family 4, and a representative of Family 1 was diagnosed with supernumerary mandible premolar. The authors suggested that the mutations may be responsible for failure to cleave aggrecan within condyle cartilage, which could explain abnormal mandibular growth [75]. In addition, an infrequent variant (Gly1121Ser1) of *ARHGAP21* was identified as a novel gene responsible for MP occurrence. According to the authors, the regulation of *ARHGAP21* by bone morphogenetic factors may explain its involvement in the etiology of this disease [109]. The *MYO1H* gene, encoding myosin 1H, has been regarded as a candidate gene in MP etiopathogenesis. Research performed with the usage of zebrafish knockdown models confirmed the participation of variant of rs3825393, C > T in abnormal mandible development [76].

A recent study comprehensively researched both sagittal and vertical skeletal malocclusion. Küchler et al. examined SNPs within genes related to bone and cartilage development and reported a positive correlation between rs3934908 (C > T) in *SMAD6*, a negative regulator of BMP signaling and MP occurrence. In addition, the correlation between the skeletal class III malocclusion phenotype and rs708111 (A > G) in *WNT3A* (wnt-3a protein) was observed, but the involvement of this mutation in the skeletal class II and III phenotypes remains unclear [110]. A summary of candidate genes involved in MP etiopathogenesis is presented in Table 2.

## 6. Contribution of Animal Models in Orofacial Clefting Research

Throughout the years, animal models have helped us to understand molecular events that lead to the development of craniofacial structures [111,112,113]. The identification of the roles played by specific pathways has provided great insight into physiological processes and has helped to target potential candidate genes in which mutation might lead to developmental defects. The modeling of nonsyndromic cleft palate NSCP is crucial for the understanding of its etiopathology, thus expanding our knowledge about developing palates.

Mice are widely used to study human diseases, including NSCP. Methods involve using transgenic mice with patient-derived mutations and a loss-of-function approach. Overall scientific preference towards murine models for humane orofacial defects originates from not only the significant anatomical resemblance but also the largely conservative molecular events leading to the formation of craniofacial structures relative to human development. However, the model has its drawbacks. The intrauterine development of the mouse palate drastically impedes access to it, making it difficult to manipulate and observe in real time.

Zebrafish have been used to address some of those issues and has emerged as a promising model of orofacial development and disease. Despite the anatomical differences, it has been shown that the developing palate of zebrafish is under similar genetic control as a mammalian palate, thus suggesting genetic conservation among vertebrates [114]. It is also easily accessible during development and susceptible to manipulations, including genetic ones.

The chicken was an experimental alternative for mice for many years and the main organism used in craniofacial research. As the processes underlying the development of viscerocranium are conserved among vertebrate species, the chicken allowed for their foundations to be discovered, including the discovery of neural crest cells. The use of the chicken also solves some of the problems associated with using mice as an animal model. For instance, access to and the real-time observation of a developing embryo in ovo are easier in chickens, even during the initial stages of embryogenesis. Accordingly, the chicken was seen to be a perfect model for studying the early processes of development such as gastrulation. The ability to tolerate inbreeding and lower maintenance costs are additional advantages [115].

In this section, we present the contributions of mice, zebrafish, and chicken models to research regarding orofacial clefting.

### 6.1. Zebrafish

Zebrafish, as a model, has deeply influenced our knowledge about various developmental processes. It has been validated as a prominent tool for the modeling and understanding of the etiopathological processes underlying congenital defects [114].

The functional equivalent of the mammalian palate used to model palatogenesis and developmental disorders associated with this process in zebrafish is the neurocranium. Despite the anatomical differences between the species, early molecular events are similar. The migration of cranial neural crest cells (CNCCs) from the dorsal neural tube, forming pharyngeal arches, is conserved among all vertebrates. As in humans, the frontonasal domain in both zebrafish and mice originates from the part of the initial stream of CNCCs; subsequently, remnant cells locate within the first pharyngeal arch to occupy the maxillary and mandibular domains. The zebrafish palate consists of several bones, and fusion takes place without the epithelial seam in the midline. At four days post fertilization (4 dpf), an ethmoid plate with cartilaginous paired trabeculae is formed as an early palate that will later contribute to the development of the adult palate.

#### 6.1.1. crispld2

Several meta-analyses and association studies have linked mutations in *crispld2* and orofacial clefting in humans, thus supporting *crispld2*’s role as a candidate gene for NSCP [116,117]. In zebrafish, it is anteriorly expressed, including developing craniofacial structures from 1 to 5 dpf. Gene silencing experiments using three morpholinos revealed its involvement in the early palate development of zebrafish. The morphants displayed dose-dependent developmental malformations including anterior and posterior clefts of the ethmoid plate, reductions in hypophyseal fenestra, and even the complete loss of those structures with severe reductions in trabeculae [118]. Another study showed that the knockdown of *crispld2* results in the abnormal migration of neural crest cells and increased cell death by promoting apoptosis [119]. These results suggest that *crispld2* may regulate cell migration and viability during the development of craniofacial structures.

#### 6.1.2. hdac4

Histone deacetylase-4, encoded by *hdac4*, regulates the activity of transcription factors involved in bone differentiation, cell growth, survival, and proliferation via the inhibition of the transcription, thereby acting as a nuclear co-repressor [120]. In humans, mutations in the gene are associated with nonsyndromic orofacial clefting and brachydactyly mental retardation syndrome [121,122]. The expression of *hdac4* in the developing tissues of zebrafish larvae begins at 4 hpf and persists until 6 hpf, as shown by a whole embryo RT-PCR [123]. Later, whole-mount in situ hybridization on 15 hpf embryos detected expression in the head structures, especially in the areas located posteriorly and dorsally to the developing eye. This pattern was found to overlap with the expression of the migrating cranial neural crest cell marker *pdgfra*, thus suggesting the co-expression of both genes within this cell type. At 72 hpf, expression was detected in *sox9*-expressing cartilages in pharyngeal arches and the mesenchyme around it, as well as in ethmoid plate and trabeculae. The silencing of *sox9* by a co-injection of two morpholinos targeting the exon-9 to intron-9 and exon-10 to intron-10 splicing sites resulted in facial-shortening. The palatal skeleton of morphants displayed a range of defects including a loss of cartilage in the ethmoid plate or trabeculae communis; the shortening and narrowing of tissues, notches, and holes within the ethmoid plate; and complete clefts of the palatal skeleton. The overexpression of *hdac4* was found to cause severe midline defects including cyclopia and a heavily reduced palatal skeleton, which suggests that *hdac4* is also involved in midline patterning early after gastrulation [123].

#### 6.1.3. Non-Canonical WNT Signaling

The non-canonical Wnt–PCP signaling pathway mediates tissue morphogenesis by regulating convergent extension [124]. It is crucial for both convergent extension and palate development, where it regulates cell proliferation and movement [114,125,126]. The expression of *wnt9a*, *frzb*, *wls*, *wnt5b*, and *gpc4*, components of the Wnt–PCP pathway, is detected during palatogenesis. *Wnt9a* is expressed in the oropharyngeal epithelium, whereas *frzb* is expressed in distal chondrocytes of the palate. *Wls* is present in mesenchymal tissues surrounding the palate and the epithelial lining of the mouth opening, overlapping with *wnt9a* and *wnt5b*, respectively, while *gpc4* is broadly expressed among chondrocytes and epithelium [127].

Loss-of-function studies using the mutants *wnt9a^c.116_118del^*, *frzb^c.481−487del^*, *wls186*, *pipetail* (*wnt5b*), and *knypek* (*gpc4*) confirmed the involvement of those genes in palate development. The wild-type zebrafish palate length/width (L/W) ratio was measured to be between 1.15 ± 0.08. *Wls* and *gpc4* mutants presented shorter and wider palates with L/W ratios of 0.7 ± 0.06 and 0.38 ± 0.07, respectively. *Frzb*^−/−^ embryos presented vaguely shortened palate with an L/W ratio of 0.94 ± 0.12. *Wnt9a*^−/−^ embryos had elongated and apparently narrower palates, though not significantly different from the wild type, whilst *wnt5b*^−/−^ mutants exhibited substantially shortened palates. Chondrocyte stacking involves convergence and extension process, and the defects in that event were also observed in both *wls*^−/−^ and *gpc4*^−/−^ embryos, where chondrocytes could not stack in a multi-layered structure and showed abnormal cell-shapes [127]. This stacking phenomenon was previously reported to be essential for zebrafish palate development [125,128]. Moreover, *wls*^−/−^ mutation affected the convergent extension of developing palates and resulted in significant shortening not associated with reduced cell proliferation or decreased cell survivability [127]. These results confirm the vital role of the Wnt–PCP pathway in both palate development and the etiopathology of orofacial clefting.

#### 6.1.4. irf6

**Figure 3:**
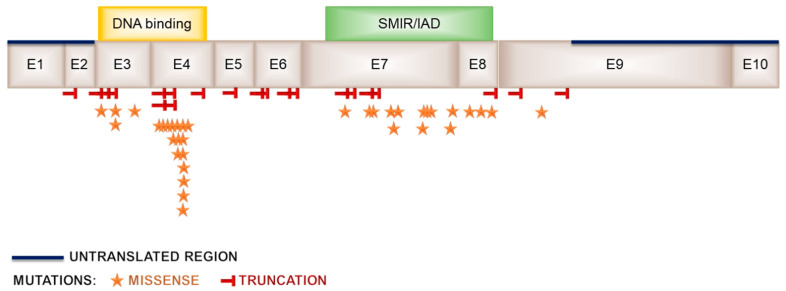
Schematic representation of mutation types within the IRF6 gene. DNA binding—winged-helix DNA binding domain; SMIR/IAD—protein binding domain.

Interferon regulatory factor 6 is a protein belonging to a family of nine proteins that share a conserved helix–turn–helix DNA binding domain (Figure 3). The majority of those proteins play mediating roles during viral infections, but the Irf6 function remained unknown for some time. Throughout the years, various patient-derived mutations have confirmed that the *irf6* is a strong candidate for contributing to both syndromic (Van der Woude syndrome) and nonsyndromic orofacial clefting, not only in humans but also other species, thus confirming its conserved role in palatal development [52,61,129]. Recently, it was shown that Irf6 is also involved in osteoblast differentiation and bone mineralization [130]. Dougherty et al. created dominant-negative mutants based on patient-derived mutations (R84C and R84H) known to contribute to orofacial clefting that were expressed under the neural-crest-specific promoter of *sox10*. Mutants were affected with clefts between the medial frontonasal and maxillary prominences, though the length of palate remained unchanged [131]. This suggests the importance of *irf6* in both the epithelium and neural crest cells in the developing palate.

#### 6.1.5. Hypoxia

Embryonic development occurs under mild hypoxic conditions. Extensive hypoxia caused by maternal smoking during the initial stages of development are strongly connected to the occurrence of CP [71]. Küchler et al. developed a protocol that allows for the examination of CP occurrence in zebrafish larvae subjected to various degrees of hypoxia. Zebrafish larvae at 8 hpf (right before CNCC migration) were placed in 30% or 50% hypoxic environments. A low level of oxygen in the water was reached by bubbling nitrogen gas, and its level was monitored using a dissolved oxygen meter. There was no significant difference between the control and test groups regarding mortality, but embryos had abnormal phenotypes depending on hypoxic conditions. They observed the hypoplasia of the anterior ethmoid plate, with a visible gap in the anterior edge forming a cleft [132]. Therefore, it is likely that the developing palate is sensitive to changes of oxygen partial pressure, a decrease in which may induce cleft. In the future, this protocol can be used to examine the pathogenic molecular patterning induced by hypoxia, possibly expanding our knowledge of the developing viscerocranium.

### 6.2. Mice

In mice, palatal structures are derived from CNCC migration, just like in zebrafish. Palate development can be divided into three processes—outgrowth, elevation, and fusion. Mesenchymal palatal shelves are covered by a layer of epithelial cells and grow vertically on both sides of the tongue. After reaching the correct length, they horizontally arrange to finally fuse above the tongue. Unlike in zebrafish, the fusion processes are dependent on the epithelium, which must be degraded. This happens via apoptosis and epithelial-to-mesenchymal transition.

#### 6.2.1. Retinoic Acid

Retinoic acid (RA) is a vitamin A derivative known to regulate organogenesis, cell growth, and differentiation during development. There is evidence suggesting that the excessive intake of vitamin A during pregnancy induces a teratogenic effect on developing palates in a dose-dependent manner, both in humans and animal models [133]. Multiple studies have shown a possible molecular mechanism of action of RA in induced clefting, but the exact etiology is not yet understood. RA interferes with BMP signaling by lowering the levels of p-SMAD2 and SMAD4 while increasing the level of SMAD7. This had a negative effect on mesenchymal cell proliferation [134]. Another study confirmed the role of RA as an Shh signaling antagonist. Mice treated with RA at E8.5 presented the abnormal expression of *Sox10*—a marker of CNCCs. The expression of this gene was found to be further altered by the administration of RA at E10.5 in the maxillary component of the first branchial arch, which later gave rise to palatal shelves. Furthermore, the downregulation of *Shh*, *Pth1*, and *Gli1* was also observed in the developing face. Additionally, a higher apoptosis rate of CNCCs was reported. The incidence of CP was found to be reduced via the ectopic administration of the Smoothened agonist (SAG) [135].

#### 6.2.2. Esrp1

Epithelial splicing regulatory protein 1 is an epithelial-specific regulator of multiple target transcripts. Its loss-of-function leads to the altered splicing of *Fgfr2*, which is linked to the CP phenotype [136]. A recent study confirmed the involvement of *Esrp1* in both palate and lip development. The RNA-Seq analysis of *Esrp1*^−/−^ ectoderm and mesenchyme provided an insight into the abnormal splicing of several molecules including FGFR2, where it was found to affect the receptor’s structure, leading to a disturbance of its physiological functions. Additionally, multiple genes were found to be up- or downregulated in the absence of *Esrp1*, including components of the WNT and SHH signaling pathways essential during palate growth [137,138]. This led to abnormal crosstalk between mesenchymal and epithelial cells leading to aberrant palate development [139].

#### 6.2.3. Sonic Hedgehog Signaling

Sonic hedgehog signaling is essential for orofacial development and mediates epithelial–mesenchymal interactions that control palatal outgrowth [10]. A recent study revealed that TGFβ and SHH together regulate the expression of tenascin-C in developing soft palates [140]. Tenascin-C is a glycoprotein of the extracellular matrix, and its expression overlays the migratory routes of cranial neural crest cells while forming future palate [141]. It mediates adhesion processes via the inhibition of fibronectin, which is also broadly expressed within palatal mesenchymal tissues and is suspected to play principal functions during palatal shelf elevation [1]. Moreover, tenascin-C is vital for regenerative processes involved in wound healing and pathological processes such as fibrosis.

There is overwhelming evidence that aberrations in SHH signaling may lead to nonsyndromic orofacial clefting [138,142,143]. *Shh* expression in epithelial rugae specifies the anteroposterior patterning of palatal shelves, thus creating a signaling center to drive mesenchymal outgrowth. Recently, Hammond et al. performed a gain of function study in which *Osr2-IresCre*; *Smo^+/M2^* embryos had ectopic SHH activity within palatal mesenchyme. In these embryos, at 13.5 dpc, mice had reduced palatal growth that resulted in significantly smaller palatal shelves that did not elevate above the tongue, even at 14.5 dpc. Later, at 15.5 dpc, embryos had a fully penetrant wide cleft of the secondary palate, while the wild-type littermates had palate already fused. The cell proliferation analysis of 13.5 dpc embryos revealed significantly reduced proliferation in both anterior and mid structures, leaving posterior structures unaffected. Besides CP, the mice had other skeletal deformities including the absence of the anterior midline premaxilla and posterior regions of the maxilla. Mandibles were significantly shorter, with noticeable ossification defects. Moreover, gene expression profiling revealed the significant upregulation of downstream targets of SHH (*Gli1* and *Ptch1*), as well as *Fox* transcription factors and several WNT/BMP antagonists such as *Sostdc1* [144]. The additional discovery of a molecular circuit *Shh–Foxf–Fgf18–Shh* by Xu et al. aided the understanding of the role of Shh signaling in regulating palatal shelf outgrowth [145].

#### 6.2.4. Pbx1/Pbx2/Pbx3

Pre-B-cell leukemia transcription factor is associated with NSCP in humans, as reported via exon sequencing data [146]. A study by Ferretti et al. focused on *Pbx* genes in craniofacial development, and the authors performed loss-of-function studies. *Pbx* genes are expressed from 8.5 to 9.0 dpc in the frontonasal process, first in the branchial arch and within the surface cephalic ectoderm and mesenchyme at E9.0. The expression increases in the epithelium of the maxillary process, medial nasal process, and the lateral nasal process by 10.5 dpc. Immunostainings confirmed the presence of PBX proteins contained by those structures, and qRT-PCR analysis confirmed *Pbx1* expression to be the strongest within those regions. Triple *Pbx1/2/3* mutants were obtained by intercrossing *Pbx1*^+/−^, *Pbx2*^+/−^, and *Pbx3*^+/−^ mice. Among the wide spectrum of mutants, they only obtained *Pbx1*^−/−^, *Pbx2*^+/−^, and *Pbx1*^−/−^; *Pbx3*^+/−^ presented fully penetrant CLP with hypoplasia of the jaw; *Pbx1*^+/−^, *Pbx2*^+/−^, and *Pbx3*^+/−^ mutants died at birth presenting isolated CP; and *Pbx1*-specific deactivation within surface cephalic ectoderm and on *Pbx2*- or *Pbx3*-deficient background resulted in orofacial clefting. There is also evidence of *Pbx* genes controlling *Wnt9b–Wnt3*, which then act upstream of *Fgf8* [147]. Research regarding the role of *Pbx1* in palate development revealed an existing *Pbx*–*Wnt*–*P63*–*Irf6* regulatory pathway that may lead to abnormal craniofacial development when disrupted [147,148].

#### 6.2.5. Tbx1/Tbx3

Tbx1 is a homeobox transcription factor involved in cellular proliferation and differentiation in oral mesenchymal and epithelial tissues, and it is necessary for epithelial shelf elongation and elevation [149,150,151]. *Tbx1* mutant mice present various clefting phenotypes including CP and submucous clefting. This variability is likely to occur due to the high number of genes that are regulated by Tbx1, as well as the genetic background of the used inbred strain. Mice lacking *Tbx1* expression in palatal shelves present the deregulated expression of *Myh3*, *Neb*, and *Gabrb3*—genes contributing to CP in affected individuals [152]. Moreover, *Tbx1*^−/−^ mice have tongue malposition and smaller mandibula, which may additionally impact the pathological process, as physical obstructions involving tongue and mandibula were previously reported in other mutants [152,153].

*Tbx3* is another T-box family gene that contributes to orofacial clefting. It is necessary for palatal shelf elevation. Mice lacking *Tbx3* expression in neural crest cells display CP, the penetration of which may depend on the genetic background of the used strain. The exact molecular mechanism of action remains unknown, but mutant mice have presented the significantly reduced expression of CP candidate gene Osr2 [154]. This, along with the co-expression of *Tbx3* and *Osr2* in mesenchymal structures, may suggest a potential role of *Osr2* in the etiopathology of CP in *Tbx3*^−/−^ mice.

### 6.3. Chicken

Their relatively big size, ease of manipulation, and accessibility (among others) are the main reasons why chickens have been utilized as models of craniofacial development for over a century. Studying this organism has provided great insight into processes underlying the formation of craniofacial structures during the development of vertebrates. Cranial neural crest cells were described for the first time in chickens [155], which were also further used to study their plasticity. Here, we describe chicken mutants that contributed or may contribute to our understanding of orofacial clefting etiopathology.

#### 6.3.1. Coloboma Mutant

Described in 1970 by Ursula Abbott, *coloboma* is a sex-linked, recessive, lethal mutation. Mutants exhibit a range of symptoms such as dwarfism and eye, limb, and craniofacial abnormalities including cleft palate that may vary in severity. The exact nature of the mutation remains uncertain, but Robb et al. attempted to map the genetic causes of developmental anomalies in the mutants. In 2011, using a 60 k SNP array, they established the genomic coordinates of a 990 Kb region on Z chromosome, confirming the sex-linked mode of inheritance [156]. Later, in a follow-up study, Robb et al. [115] focused on four genes within this region—*CDK7*, *CENPH*, *SLC30A5*, and *MRPS36*. Though the results were not conclusive, the molecular mechanism remains to be discovered and revealing it may be of great importance to our understanding of craniofacial etiopathology [115].

#### 6.3.2. Cleft Primary Palate Mutant

A chicken mutant discovered within the stock of UC Davis in 1966 and characterized by Ursula Abbott displayed a severe craniofacial phenotype caused by the abnormal development of frontonasal prominence unable to fuse with maxillary prominence, thus resulting in a cleft primary palate. Unfortunately, the mutant remained poorly studied until recently, when an attempt was made to pathogenically map it. After narrowing the search area to the selected region of GGA 11 microchromosome, it was possible to identify the frameshift mutation within the *ESRP2* gene [157]. *ESRP2* is an alternative splicing regulator for genes such as *FGFR2* [158]. *FGFR* genes are known to influence cell proliferation and differentiation during development [159]. Moreover, impaired FGF signaling relates to CLP occurrence in humans [160]. *ESRP1* is another member of the family who’s—as mentioned above—knockout in mice also leads to CLP and is lethal early after birth. Together, these data may suggest that a mutation within *ESRP2* is responsible for the *cpp* phenotype, but more must be done to understand the entire process.

## Figures and Tables

**Table 1:** Selected examples of mutations in CLP patients. This table contains data available in public databases in October 2021. * According to GeneCards data.

Chromosomal Localization	Biological Function *	Mutation	Number of Patients	Additional Phenotype	References
***MSX1*** *chr4:4,859,665-4,863,936*	Encodes a member of the muscle segment homeobox gene family. MSX1 acts as a repressor during embryogenesis and plays a role in craniofacial development, limb-formation, and odontogenesis.	Ser104stop (exon 1)	2	Tooth agenesis	[58]
***PVRL1* (*NECTIN-1*)** *chr11:119,623,408-119,729,200*	Encodes an adhesion protein that takes part in the organization of adherens junctions and tight junctions in epithelial and endothelial cells.	Trp185TGG- > TAG	4	Margarita Island ectodermal dysplasia	[59]
		Gly323GGT- > GGTT	1	Złotogór-Ogur syndrome	
***IRF6*** *chr1:209,785,617-209,806,175*	Encodes a member of the interferon regulatory transcription factor family. Determines keratinocyte proliferation–differentiation switch (vital in appropriate epidermal development).	c.250C > T; p.Arg84Cys.	Family A–1 affected	N/A	[60]
		c.1060 + 1G > T; p. N/A	Family B–1 affected	N/A	
		c.379delG; p.Gly127Valfs *43	Family C–1 affected	N/A	
		c.39G > A; p.Trp13 *	Family D–4 affected	Upper lateral incisor bilateral lower lip pits extra right maxillary molar	
		c.254G>; p.Cys85Phe	Family E–3 affected	N/A	
		c.165delC; p.Ile56Phefs*7	Family F–1 affected	N/A	
		c.1289_1297del; p.Asp430_Ile432del	Family G–5 affected	Presence of a single pit on the lower lip in the proband	
		c.26G > A; p.Arg9Gln	2	Absence of both upper second incisors	[61]
***VAX1*** *chr10:117,128,520-117,138,301*	Encodes a homeo-domain containing proteins from a class of homeobox transcription factors. Plays a role in regulation of body development and morphogenesis.	c.3890G > A; p.Ala201Thr	Family 1–1 affected	N/A	[62]
		c.3828G > C; p.Arg180Pro	Family 2–2 affected	N/A	
		c.1676C > T; p.Pro92Leu	Family 3–3 affected	N/A	
***TBX22*** *chrX:80,014,753-80,031,774*	Encodes member of genes family that share a common DNA-binding domain, the T-box. Major determinant for palatogenesis.	IVS6-1G > C splice site mutation	Family 1–6 affected (X-linked cleft palate) Family 2–2 affected (cleft lip, cleft palate, cleft alveolar) 200 healthy controls	N/A	[63]
***CDH1*** *chr16:68,737,292-68,835,537*	Encodes a member of cadherin superfamily that is involved in mechanisms regulating cell–cell adhesions, mobility, and proliferation of epithelial cells.	c.687 + 1G > A	Family 1–3 affected	Hereditary diffuse gastric cancer (HDGC)	[64]

**Table 2:** Selected examples of mutations in MP patients. The table contains data available in public databases in October 2021. * According to GeneCards data.

Chromosomal Localization	Biological Function *	Mutation	No of Affected Patients	Additional Phenotype	References
***MATN1*** *1p36*	Cartilage matrix protein. Major component of the extracellular matrix of non-articular cartilage.	7987 G > A 8572 C > T	164 patients	N/A	[98]
***EPB41*** *1p36*	Together with spectrin and actin, plays critical role in formation of erythrocyte membrane skeleton.	rs2788890 rs2788888 rs2254241 rs2249138	158 patients	N/A	[99]
***ADAMTS1*** *21q21.3*	Cleaves aggrecan, a cartilage proteoglycan, and may be involved in its turnover.	742 I > T rs2738 rs229038	9 patients 230 patients	N/A	[108]
		176 C > A	Family 1 (3 patients)	Supernumerary tooth (mandible premolar)	[75]
		670 C > G 2 of 4 affected patients had additional COL1A2 mutation	Family 2 (4 patients)	Osteogenesis imperfecta, Dentinogenesis imperfecta	
		670 C > G	Family 3 (1 patient)	N/A	
		670 C > G WNT10B additional mutation	Family 4 (1 patient)	N/A	
***ARHGAP21*** *10p12.1*	Regulates the ARP2/3 complex and F-actin dynamics at the Golgi apparatus through the control of CDC42 activity.	3361 G > A	59 patients	N/A	[109]
***COL2A1*** *12q13*	Encodes the alpha-1 chain of type II collagen, a fibrillar collagen found in cartilage and the vitreous humor of the eye.	rs1793953 G > A	211 patients	N/A	[103]
***MYO1H*** *12q24.11*	Actin-based motor molecule with ATPase activity. Serves in intracellular movements.	rs3825393 C > T	199 patients	N/A	[76]
***SMAD6*** *15q22.31*	Involved in the mesodermal commitment pathway and BMP signaling. Associated with aortic valve disease 2 and craniosynostosis 7.	rs3934908 C > T	50 patients	N/A	[110]

## Data Availability

Not applicable.

## Supplementary Materials

The following are available online at https://www.mdpi.com/article/10.3390/ijms23020953/s1.
