# Supplementary material for: Orofacial Cleft and Mandibular Prognathism—Human Genetics and Animal Models
Source: Int J Mol Sci. 2022 Jan 16;23(2):953. doi: 10.3390/ijms23020953 (PMC8779325; doi:10.3390/ijms23020953)
Supplement: Supplementary file 1 [file ijms-23-00953-s001.zip › ijms-1480196-supplementary.pdf]

Measurement	Norm	Value
SNA (°)	82.0 ± 3.0	79.2
SNB (°)	80.0 ± 3.0	81.3
ANB (°)	2.0 ± 2.0	-2.1
Wits (mm)	0.0 ± 2.0	-11.7
SNPg (°)	81.0 ± 3.0	81.9
NSBa (°)	132.0 ± 4.0	137.0
Gn-tgo-Ar (°)	122.0 ± 7.0	128.7
NL-NSL (°)	8.0 ± 4.0	12.3
ML-NSL (°)	28.0 ± 5.0	41.1
ML-NL (°)	20.0 ± 7.0	28.8
H (°)	9.0 ± 3.0	3.3
1+:1- (°)	133.0 ± 8.0	128.7
1+:NA (°)	21.0 ± 4.0	22.4
1-:NB (°)	24.0 ± 4.0	31.0
Nose-Lip (°)	110.0 ± 7.0	104.6
Pg:NB (mm)	2.3 ± 2.0	1.2
1+:NA (mm)	3.7 ± 2.0	5.2
1-:NB (mm)	3.8 ± 5.0	7.0
Index	80.0 ± 7.0	74.7

Table S1. Cephalometric analysis according to Segner and Hasund presenting patients diagnosed with skeletal class III malocclusion before treatment. S – Sella; N – Nasion; A – Subspinale; B – Supramentale; Wits - distance between point A and B projected on occlusal plane; Pg – Pogonion; Ba – Basion; Gn – Gnathion; tgo – Gonion; Ar – Articulare; NL – Spina nasalis anterior-Pterygomaxillare line; NSL – Nasion-Sella line; ML – Gnathion-Gonion line; H – angle between WPg-UL line (WPg – Skin point Pogonion; UL – Upper lip point) and NB line; 1+:1- - Angle between lines through long axis of upper and lower central incisors; 1+:NA/1-:NB (°) – Angle between line through long axis of upper/lower central incisor and NA/NB line; 1+:NA/1-:NB (mm) – Distance between tip of upper/lower central incisor and NA/NB line; Nose-Lip – angle between ctg (Columella), Sn (Subnasale) and UL; Pg:NB – Length between Pg and NB line; Index – Ratio of the length of segments N-Sp’ (Spina’) and Sp’-Gn.
